# PREDICTORS OF CONTINUED ENGAGEMENT IN ONE’S FAVORITE ACTIVITY DURING THE COVID-19 PANDEMIC

**DOI:** 10.1093/geroni/igae098.2891

**Published:** 2024-12-31

**Authors:** Jeehoon Kim, Ji Hyun Lee

**Affiliations:** Idaho State University, Pocatello, Idaho, United States; Montana State University, Bozeman, Montana, United States

## Abstract

Active engagement is one of the important pillars in successful aging (Rowe & Kahn, 1997), however less is known about one’s favorite activity, a key source of enjoyment and meaning, in later life. In the context of higher constraints imposed during the pandemic, continuing one’s favorite activity can play a pivotal role in maintaining well-being for older adults. The current study aimed to examine which factors are associated with sustaining engagement in one’s favorite activity in 2020. Data comes from Round 10 of National Health and Aging Trends Study. Participants (n= 4,389, Age M(SD)=78(6), 55% female, 83 % White) answered, during last year, whether they were able to participate in their favorite activity previously reported at Round 5 (84% yes). Survey weighted logistic regression models were performed stratified by gender. We found that women who reported health better (OR= 1.28, p=.018) and engaged in vigorous physical activity (OR=1.96, p =.007) were more likely to continue their favorite activity. For men, younger age (OR=.97, p=.017), social engagement (OR=1.22, p =.011) and physical activity participation (walking OR=1.91, p=.014; vigorous OR=2.07, p=.001) were positively, but depressive symptoms (mild OR=.57, p =.006; major OR=.50, p=.028) were negatively linked to continuing in favorite activities. The findings show that engagement in physical activity supports continued enjoyment of one’s favorite activity; social engagement and mental health are also protective factors for men. Implications for how to support older women and men’s continued activity engagement will be discussed.

